# Healthy Eating Is More than the Foods You Eat: Eating Practices of Mothers with and Without a History of Gestational Diabetes Mellitus †

**DOI:** 10.3390/healthcare13212792

**Published:** 2025-11-04

**Authors:** Mélissa Bélanger, Charlotte Simoneau, Julie Perron, Simone Lemieux, Julie Robitaille

**Affiliations:** 1 School of Nutrition, Université Laval, Québec, QC G1V 0A6, Canada; melissa.belanger.5@ulaval.ca (M.B.); simone.lemieux@fsaa.ulaval.ca (S.L.); 2 Centre Nutrition, Santé et Société (NUTRISS), Institut sur la Nutrition et les Aliments Fonctionnels (INAF), Université Laval, Québec, QC G1V 0A6, Canada; charlotte.simoneau.1@ulaval.ca (C.S.); julie.perron@fsaa.ulaval.ca (J.P.); 3 Endocrinology and Nephrology Axis, CHU de Quebec Research Center, Québec, QC G1V 4G2, Canada

**Keywords:** diabetes, gestational, dietary guidelines, eating behavior, diet quality, food, processed, food environment, anthropometry, cardiometabolic risk, chronic disease

## Abstract

**Background/Objectives**: Canada’s Food Guide 2019 includes advice such as “Cook more often” and “Eat meals with others”, which are considered healthy eating practices. However, mothers with a history of gestational diabetes mellitus (GDM) may face specific barriers to adopting healthy eating practices. This study aimed to compare eating practices between mothers with (GDM+) and without (GDM−) a history of GDM, and to explore the associations between eating practices, diet quality, and the anthropometric and cardiometabolic profile of these mothers. **Methods**: The cross-sectional study was conducted in Quebec (Canada) between 2012 and 2017. Eating practices were assessed using a self-administered questionnaire. Diet quality was evaluated by the Healthy Eating Food Index 2019 through a validated food frequency questionnaire. Weight, height, and waist circumference were measured, and body composition was obtained by absorptiometry. **Results**: Data from 105 GDM+ and 38 GDM− mothers were analyzed (mean age 37.5 years ± 4.9). GDM+ mothers were more likely to prepare a greater proportion of dinners (≥1 per week) using pre-prepared or processed foods than GDM− mothers (49.0% vs. 34.2%; *p* = 0.016). Among GDM+ mothers, those who prepared ≥1 dinners per week using pre-prepared or processed foods showed lower adherence to the “Whole-grain foods” (1.1 ± 0.8 vs. 1.9 ± 1.2; *p* = 0.002) and “Sodium” (4.9 ± 2.0 vs. 5.8 ± 2.0, *p* = 0.013) recommendations, had a higher percentage of total body fat (37.5% ± 7.6 vs. 34.0% ± 7.7; *p* = 0.041), a higher waist circumference (91.6 cm ± 13.9 vs. 87.1 cm ± 16.3; *p* = 0.030), and a higher glycated hemoglobin (5.6% ± 0.5 vs. 5.5% ± 0.3; *p* = 0.025) than those who used less pre-prepared or processed foods. **Conclusions**: GDM+ mothers were more likely than GDM− mothers to prepare dinners using pre-prepared or processed foods, an eating practice associated with less favorable components of diet quality and some altered anthropometric and cardiometabolic variables. Further investigation into the factors influencing cooking from scratch within this population is warranted.

## 1. Introduction

In alignment with the statement “Healthy Eating is More Than the Foods You Eat”, Canada’s Food Guide (CFG) 2019 includes advice such as “Be mindful of your eating habits”, “Enjoy your food”, “Cook more often”, and “Eat meals with others” [1]. Beyond the content of our plates, the context in which food is consumed has garnered increasing interest, referred to as eating practices [2]. In the general population, these eating practices have been linked to a better diet quality and, ultimately, improved overall health [2,3,4,5]. However, like other high-risk populations, mothers with a history of gestational diabetes mellitus (GDM) may encounter specific barriers to adopting healthy eating practices, such as a lack of information about healthy eating after their complicated pregnancy [6,7,8], which in turn may influence the food environment of their high-risk offspring [9].

GDM is defined as hyperglycemia with onset or first recognition during pregnancy [10]. In Canada, the prevalence of GDM was 10.4% in 2019, according to the most recent data—a rate that has doubled in one decade [11]. Mothers with a history of GDM (GDM+) are at an increased risk of developing chronic diseases in the years following delivery [12,13,14,15]. They are ten times more likely to develop type 2 diabetes (T2D) and twice as likely to suffer from cardiovascular diseases (CVD) compared to mothers with no history of GDM (GDM−) [12,13].

Knowledge about eating practices among mothers with a history of GDM is scarce. According to a previous study by our group, based on the Theory of Planned Behavior, the disapproval of a family member other than the partner was a subjective norm associated with a lower intention to adopt a healthy diet among GDM+ mothers [6]. Social influence also appears to be an important determinant of diet among individuals at risk for T2D or CVD [16,17], highlighting the importance of studying the eating context specifically among GDM+ mothers.

To our knowledge, unlike diet quality, eating practices have not yet been explored in mothers with a history of GDM. Multiple studies showed that a better diet quality was associated with a more favorable anthropometric and cardiometabolic profile among GDM+ mothers [14,18,19,20,21]. According to a recent scoping review on the association between specific dietary patterns and cardiometabolic outcomes in GDM+ mothers, providing simple and adaptable dietary guidance could make any proposed intervention easier to implement and improve adherence within this population [19]. Since GDM+ mothers often struggle to maintain a healthy diet after delivery [7,22,23,24,25], it is essential to analyze their diet in a more comprehensive way, focusing on the “how” of eating rather than just the “what”. Eating practices offer an opportunity for developing more practical and specific dietary recommendations for these at-risk mothers [7,26]. The first objective of this study was to compare eating practices between GDM+ and GDM− mothers. The second objective was to explore the associations between eating practices and (1) diet quality, and (2) anthropometric and cardiometabolic profiles of GDM+ and GDM− mothers.

## 2. Materials and Methods

### 2.1. Study Population

Recruitment of the participants for the GDM2 study took place between 2012 and 2017. The aim of this cross-sectional study was to evaluate the impact of GDM on maternal and offspring health. A total of 143 mothers (105 GDM+ and 38 GDM−) enrolled in this study. More details on the study design can be found elsewhere [6,27]. Briefly, mothers were recruited through medical records from the two main hospitals with a neonatal care unit in Quebec City (*Hôpital Saint-François d’Assise* and *Centre Hospitalier de l’Université Laval*), the provincial health plan registry (*Régie de l’assurance maladie du Québec*), emails sent to the Laval University community, and posts on healthcare websites and social networks. French-speaking mothers from the Quebec City metropolitan area aged ≥18 years who carried a pregnancy between 2003 and 2013 were invited to participate. Those who were pregnant at the time of the study or had pre-existing diabetes (type 1 or type 2) were excluded. Mothers were invited for a single visit (lasting 3 to 4 h) at the Institute of Nutrition and Functional Foods (INAF) in Quebec City, Canada, during which all data were collected. GDM was diagnosed between 24 and 28 weeks of gestation using the sequential two-step approach [28,29,30]. Mothers were recruited on average 6.1 ± 2.4 years after their last pregnancy complicated by GDM. Written consent was obtained from all participants, and ethical approval was obtained from the *Université Laval* Ethics Committee (2011-196-A-4 R-3) and the *Centre Hospitalier Universitaire de Québec* Ethics Committee (2015–2031, B14-07-2031-21). This study was registered in the Clinical Trials.gov registry (NCT01340924).

### 2.2. Eating Practices

Eating practices were collected through a self-administered questionnaire, which mothers completed with a provided electronic device during their visit to the research center. If needed, mothers could receive assistance with completing the questionnaire. Since data collection was conducted before the publication of the CFG 2019, the questions that better reflect the practices targeted in the guide were included in this study. Three categories of eating practices were documented as part of the GDM2 study: “Be mindful of your eating habits”, “Eat meals with others” and “Cook more often” [1].

One question was related to the “Be mindful of your eating habits” practice: “How many times have you had meals in front of/with a screen in the past seven days?”. This question was open-ended (i.e., answers given as the number of breakfasts, lunches, and dinners per week). A “screen” was defined as television, computer, gaming console, iPad, etc. Another open-ended question allowed the analysis of “Eat meals with others” practice: “How many times have you had family meals in the past seven days?”. Family meals were defined as meals taken with children and at least one parent. The “Cook more often” practice was assessed using this open-ended question: “In the past seven days, how many times was dinner (out of a total of 7)…” (1) “…cooked from scratch?”, (2) “…cooked with pre-prepared or processed foods?”, (3) “…ready-to-eat?” or (4) “…take-out/restaurant?”. Some examples were provided to help mothers select the best answer. Dinners cooked with pre-prepared or processed foods were defined with examples such as the use of marinated meat or fish, packaged sauces, or ready-to-cook items from the grocery store. Ready-to-eat dinners were defined as frozen meals, canned soup or ready-to-eat lasagna, as examples.

### 2.3. Diet Quality

Mothers completed a validated web-based food frequency questionnaire (web-FFQ) with a provided electronic device during their visit to the research center [31]. This tool is an online self-administered quantitative FFQ that has been validated to assess dietary intakes over the last month among the French-speaking Canadian population [31]. Food, nutrient, and energy intake data were derived from a food composition database developed and validated for this web-FFQ, based on the Nutrition Data System for Research (software version 4.03, Food and Nutrient Database 31, Minneapolis, MN, USA) and the Canadian Nutrient File (CNF, version 2007b, Ottawa, ON, Canada) [31]. The diet quality was evaluated by the Healthy Eating Food Index (HEFI)-2019 and its ten components [32]. HEFI-2019 is a score of adherence to the 2019 CFG recommendations [32]. Seven of the HEFI-2019 components are “adequacy” components (i.e., greater adherence is defined by greater relative consumption): Vegetables and fruits, Whole-grain foods, Grain foods ratio, Protein foods, Plant-based protein foods, Beverages, and Fatty acids ratio [32]. HEFI-2019 also includes three “moderation” components (i.e., greater adherence is defined by lower relative consumption) that relate to nutrients of concern: Saturated fats, Free sugars, and Sodium [32].

Since healthy eating practices can be associated with the use of less calorie-dense foods [26,33,34], adjusting for energy intake in our analyses helps connect the “what” and the “how” of eating, provided that energy intake is accurately reported. An additional adjustment for the reporting status allows for handling misreporting [35], which was determined using the ratio of self-reported energy intake to predicted energy requirements [36,37]. Mothers with a ratio of <0.78, 0.78 to 1.22, or >1.22 were categorized as under-, plausible, and over-reporters, respectively [36].

### 2.4. Anthropometric and Cardiometabolic Profile

Mothers’ weight was measured in light clothes and without shoes using a calibrated balance to the nearest 0.1 kg (Tanita BC-418, Tanita Corporation of America Inc., Arlington Heights, IL, USA). Their height was measured with a stadiometer to the nearest millimeter. Body mass index (BMI) was calculated (kg/m^2^). Waist circumference was measured twice, under the clothes, following a standardized procedure at the midpoint between the iliac crest and the last palpable rib [38]. Mothers’ body composition and fat distribution were also measured using a dual-energy X-ray absorptiometry scanner (DXA, GE Lunar Prodigy Bone Densitometer, GE Healthcare Lunar, Madison, WI, USA). Two variables were considered: total fat mass (%), and android fat mass (%).

Fasting blood samples were collected, and a 75 g, 2 h oral glucose tolerance test (OGTT) was performed. During this test, blood samples were collected at −15, 0, 15, 30, 60, 90, and 120 min to measure glucose and insulin concentrations. Plasma glucose was measured enzymatically, and insulin was measured by radioimmunoassay [14]. Glycated hemoglobin (HbA1C) was determined using the Cobas Integra 800 analyzer (Roche Diagnostics, Switzerland) standardized to the National Glycated Hemoglobin Standardization Program [39]. HbA1C reflects the average glucose level over the past 2–3 months. Homeostasis model assessment of insulin resistance (HOMA-IR) was calculated from fasting glucose and insulin concentrations to evaluate insulin resistance [40]. Lipids were measured using automated enzymatic methods [41]. The cholesterol ratio was obtained by dividing total cholesterol (mmol/L) by HDL cholesterol (mmol/L); a higher ratio indicates a higher risk of CVD [42].

### 2.5. Maternal Data

Sociodemographic characteristics were collected through self-administered questionnaires. Mothers were asked about their age, ethnicity, household annual income, highest level of education, age of their youngest child (classified as preschool-aged if ≤5 years old or school-aged if 6–12 years old), and number of children. Mothers were not asked about their gender. Of note, the term “mothers”, as used in this manuscript, refers to the parent whose biological sex is female and who has given birth to one or more children.

Mothers’ lifestyle habits were also used as covariates for the second objective of this study (physical activity, smoking status, and alcohol consumption). Physical activity was objectively assessed using ActiGraph GT3X triaxial accelerometers (ActiGraph, Pensacola, FL, USA). Mothers were instructed to wear it over the hip on an elasticized belt during 7 consecutive days after their visit to the research center. More details on the use of accelerometers in the GDM2 study can be found elsewhere [43]. The number of minutes of moderate-to-vigorous physical activity per day was extracted. Current smoking status (yes or no) and average alcohol consumption (number of consumptions per week) were also collected though a self-administered questionnaire during their visit to the research center.

### 2.6. Statistical Analyses

Mothers’ characteristics according to their history of GDM were compared using Chi-square tests (or Fisher exact tests) for categorical variables. Results on eating practices were presented as dichotomous variables; categories were created according to the mean of each eating practice. For example, the number of dinners cooked from scratch was categorized as <5 dinners per week or ≥5 dinners per week (mean of 5.3 ± 1.3 per week).

Eating practices were compared between GDM+ and GDM− mothers using log-binomial regression, adjusting for age, household annual income, highest maternal level of education, age of the youngest child, and number of children [44,45,46,47]. Further adjustment for energy intake and reporting status was also performed. Eating practices that differed between the two groups were selected for further analyses. Generalized linear models (GLMs) were performed to examine the association between these eating practices and diet quality (HEFI-2019) among GDM+ and GDM− mothers separately, adjusting for the same covariates previously described.

In addition, GLMs were performed to examine the relationship between eating practices and anthropometric and cardiometabolic measures in GDM+ and GDM− mothers separately. Two models were used: Model 1, which adjusted for potential covariates (age, household annual income, highest maternal level of education, moderate-to-vigorous physical activity, smoking, and alcohol consumption), and Model 2, which further adjusted for energy intake and reporting status. These covariates were selected based on their influence on mothers’ eating practices, anthropometric measures, and cardiometabolic measures according to the current literature [45,46,47,48,49]. Variables that were not normally distributed were transformed using the Box–Cox procedure when necessary. The statistical software SAS OnDemand for Academics (SAS Studio Version 3.82) was used for the analyses. Results presented in this study are from exploratory analyses and were not pre-registered.

## 3. Results

The mean age of mothers was 37.5 ± 4.9 years, and more than 90% identified as White. Mothers’ characteristics according to prior GDM status are presented in Table 1. GDM+ mothers were older than GDM− mothers; with 37.5% of GDM+ mothers being 40 years old or older, compared to 29.0% of GDM− mothers. Other sociodemographic characteristics were similar between GDM+ and GDM− mothers.

### 3.1. Eating Practices

Eating practices are shown in Table 2. GDM+ and GDM− mothers shared a similar proportion of family meals and meals eaten in front of a screen. However, GDM+ mothers were more likely to prepare a greater proportion of dinners (≥1 per week) using pre-prepared or processed foods compared to GDM− mothers (49.0% vs. 34.2%, *p* = 0.016), after adjusting for age, household annual income, highest maternal level of education, age of the youngest child, and number of children. Similar results were obtained after further adjustment for energy intake and reporting status. Other methods of preparation (number of dinners cooked from scratch, ready-to-eat, or purchased at a take-out restaurant) were similar between GDM+ and GDM− mothers.

### 3.2. Diet Quality

Among GDM+ mothers, those who prepared ≥1 dinners per week using pre-prepared or processed foods had a mean energy intake of 2287 ± 641 kcal/d vs. 2099 ± 460 kcal/d for those who prepared no dinner of this type (*p* = 0.681, Table S1). Similar results were observed among GDM− mothers (2737 ± 919 kcal/d vs. 2404 ± 688 kcal/d, *p* = 0.371, Table S2). The proportion of under-, plausible, and over-reporters was not different between the two groups for GDM+ mothers (*p* = 0.386; Table S1), but was different for GDM− mothers with more over-reporters among those who prepared ≥1 dinners per week using pre-prepared or processed foods compared to those without dinners using pre-prepared or processed foods (53.9% vs. 44.0%, respectively; *p* = 0.030; Table S2).

**Figure 1:**
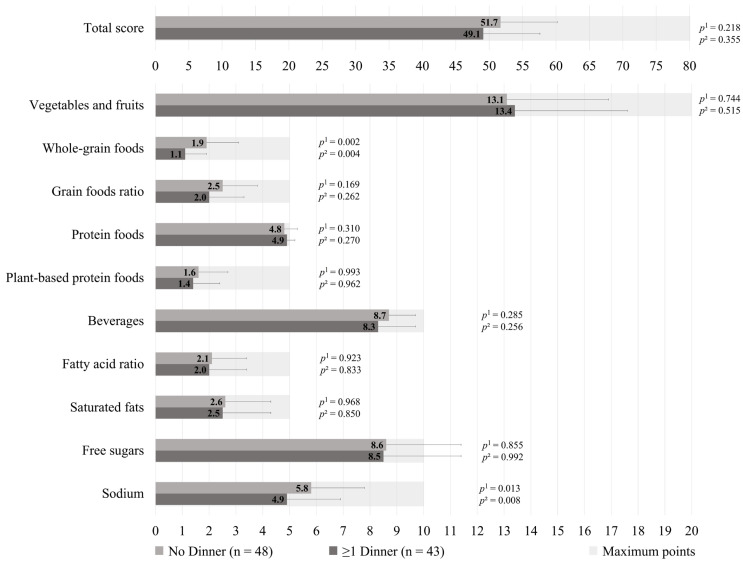
Healthy Eating Food Index—2019 of GDM+ mothers according to the number of dinners using pre-prepared or processed foods per week. Note: GDM+: history of gestational diabetes. ^1^ Adjustment for age, household annual income, highest maternal level of education, age of the youngest child, and number of children. ^2^ Further adjustment for energy intake and reporting status.

**Figure 2:**
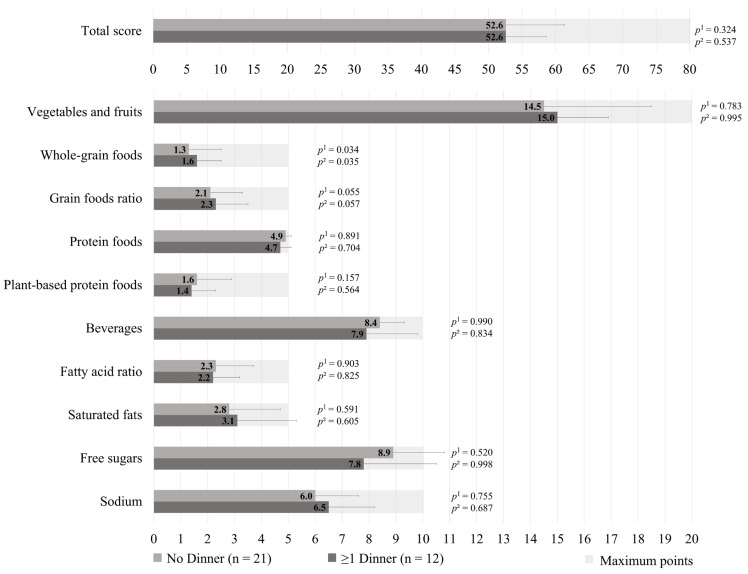
Healthy Eating Food Index—2019 of GDM− mothers according to the number of dinners using pre-prepared or processed foods per week. Note: GDM−: no history of gestational diabetes. ^1^ Adjustment for age, household annual income, highest maternal level of education, age of the youngest child, and number of children. ^2^ Further adjustment for energy intake and reporting status.

The HEFI-2019 score was similar between those who prepared ≥1 dinners per week vs. no dinner using pre-prepared or processed foods among GDM+ mothers (49.1 ± 8.5 vs. 51.7 ± 8.5, respectively; *p* = 0.218), as shown in Figure 1. More specifically, the first group adhered less to the “Whole-grain foods” (1.1 ± 0.8 vs. 1.9 ± 1.2, respectively; *p* = 0.002) and “Sodium” (4.9 ± 2.0 vs. 5.8 ± 2.0, respectively, *p* = 0.013) components of the HEFI-2019 score compared to the latter. Among GDM− mothers, those who prepared ≥1 dinners per week vs. no dinner using pre-prepared or processed foods had a similar HEFI-2019 score (52.6 ± 6.0 vs. 52.6 ± 8.7, respectively; *p* = 0.324), as shown in Figure 2. Conversely to GDM+ mothers, GDM− mothers who prepared ≥1 dinners per week using pre-prepared or processed foods adhered more to the “Whole-grain foods” (1.6 ± 0.9 vs. 1.3 ± 1.2, respectively; *p* = 0.034) component compared to those who prepared no dinner of this type. The HEFI-2019 results are also presented in Supplementary Table S3 for GDM+ mothers and in Supplementary Table S4 for GDM− mothers.

### 3.3. Anthropometric and Cardiometabolic Profile

Anthropometric and cardiometabolic profiles among GDM+ mothers according to the proportion of dinners per week using pre-prepared or processed foods are shown in Table 3. Those who prepared ≥1 dinners per week using pre-prepared or processed foods had a higher percentage of total body fat (37.5% ± 7.6 vs. 34.0% ± 7.7; *p* = 0.041), a higher waist circumference (91.6 cm ± 13.9 vs. 87.1 cm ± 16.3; *p* = 0.030) and a higher glycated hemoglobin (5.6% ± 0.5 vs. 5.5% ± 0.3; *p* = 0.025) than those who prepared a lower proportion, after adjustment for age, household annual income, highest maternal level of education, moderate-to-vigorous physical activity, smoking, and alcohol consumption. A similar result for glycated hemoglobin was obtained after further adjustment for energy intake and reporting status. However, differences in total body fat and waist circumference were no longer significant (*p* = 0.090 and *p* = 0.088, respectively). Among GDM− mothers, as presented in Table 4, no significant anthropometric and cardiometabolic differences were observed between those who prepared ≥1 dinners per week using pre-prepared or processed foods and those who prepared no dinners of this type.

## 4. Discussion

Results of this study showed that mothers with a history of GDM adopted less favorable eating practices compared to mothers without a history of GDM according to CFG 2019 recommendations [1]. Specifically, a greater proportion of GDM+ mothers cooked dinners using pre-prepared or processed foods compared to GDM− mothers. This eating practice was associated with less favorable components of diet quality and with altered anthropometric and cardiometabolic measures among GDM+ mothers.

We showed that GDM+ mothers were more likely to prepare a greater proportion of dinners using pre-prepared or processed foods than GDM− mothers. Half (49%) of GDM+ mothers cooked one or more dinners per week using pre-prepared or processed foods, compared to a third (34%) of GDM− mothers. To our knowledge, this is the first study focusing on eating practices among mothers with a history of GDM. GDM+ mothers represent a unique high-risk population for T2D. They encounter multiple barriers to healthy eating that are common to all mothers—such as lack of time, children’s food preferences, and work schedule [26,50,51]—but they also encounter specific barriers: no systematic postpartum follow-up after a pregnancy complicated by GDM in Canada’s healthcare system, lack of information about healthy eating after childbirth, lack of direct impact of the maternal diet on the child’s health (unlike during pregnancy), and possible return to a sub-optimal diet similar to the pre-pregnancy diet [6,8,25,52,53,54]. Numerous studies have also shown that a large proportion of GDM+ mothers are unaware that this pregnancy complication is associated with an increased risk of T2D and CVD after delivery, which may partly explain why they do not consistently adopt healthy lifestyle habits [55,56,57]. The proportion of cooked dinners using pre-prepared or processed foods per week among GDM− mothers (7.5% ± 14.0) was similar to findings from a study of 150 ethnically and socioeconomically diverse families with young children in the United States: 7% of meals were partly home-cooked [58]. In comparison, among GDM+ mothers, the proportion of dinners using pre-prepared or processed foods per week was nearly doubled (14.1% ± 18.3).

Globally, our results highlight the complexity of healthy eating, where both the “what” and the “how” of eating interact synergistically and are difficult to separate. Home-cooked meals are known to be more nutritious than ready-to-eat meals or those purchased at a takeout restaurant [4,5,59]. However, considerable variability exists in how meals can be cooked [58,60]. It is likely that cooking more frequently with pre-prepared or processed foods is associated with some unfavorable dietary components, such as a higher energy intake and a lower diet quality [33,61]. Indeed, GDM+ mothers who prepared ≥1 dinners using pre-prepared or processed foods per week had less favorable components of diet quality (i.e., a lower consumption of whole grains and a higher consumption of sodium) than those without dinners using pre-prepared or processed foods. Consistent with our findings, some studies showed that cooking less often from scratch was associated with some unfavorable dietary components, such as a lower vegetable and fruit intake and a higher ultra-processed food consumption [58,60,62].

The results of our study also showed that, among GDM+ mothers, those who prepared ≥1 dinners per week using pre-prepared or processed foods had a higher percentage of total body fat, a higher waist circumference, and a higher HbA1C than those without dinners using pre-prepared or processed foods. Since pre-prepared and processed foods are generally more calorie-dense than whole foods [33], we made further adjustments for energy intake and reporting status, and observed that the results were attenuated. However, differences in HbA1C remained significant even after these adjustments. Since GDM+ mothers are ten times more likely to develop T2D later in life compared to GDM− mothers [13] and that even small changes in HbA1C matter regarding cardiometabolic risk [10,63], this result is important from a clinical standpoint.

In parallel, in the context of this study, the anthropometric and cardiometabolic profile among GDM− mothers between those who prepared ≥1 dinners per week vs. no dinner using pre-prepared or processed foods were similar. GDM− mothers who prepared ≥1 dinners per week using pre-prepared or processed foods had a similar HEFI-2019 and a favorable dietary component (i.e., a higher consumption of whole grains) compared to those without dinners using pre-prepared or processed foods, which may explain the lack of association with anthropometric and cardiometabolic variables. Pre-prepared and processed foods fall within a broad and heterogeneous category [58,60]. It is possible that GDM+ mothers make less optimal choices within this category compared to GDM− mothers, which aligns with findings related to the HEFI-2019 components. This hypothesis highlights an opportunity for nutrition education targeting GDM+ mothers, who may lack the time or skills to prepare meals from scratch [22]. Providing them with guidance to make healthier choices among pre-prepared or processed foods—while respecting their family’s realities—could support more effective dietary interventions [19,25].

The limitations of this study include the fact that the socioeconomic status of the GDM2 study was high, despite efforts to recruit from a more vulnerable area of Quebec City, which may limit the generalizability of the results. The limited number of GDM− mothers in this study may increase the risk of false-negative results. Since our results were obtained from cross-sectional data, our findings should be interpreted with caution and confirmed in longitudinal studies. The Canadian Eating Practices Screener remains the validated tool for measuring eating practices according to the CFG 2019; however, this screener was not available at the time of the study [1,2]. The method of preparation for other meals of the day (i.e., breakfast and lunch) was also not documented. Culturally, dinner remains the most important meal of the day in Canada—the one most likely to be cooked and shared with family [45,64]. The strengths of this study include the exclusion of other types of diabetes or pregnancy complications associated with various outcomes for mothers, compared to GDM [65,66]. The presence of a control group also enabled a comparison of eating practices between GDM+ and GDM− mothers. One of the main strengths of this study was our approach to studying diet in a more global way, by including the analysis of eating practices among GDM+ mothers. Finally, the OGTT provides a more comprehensive glycemic profile, while the DXA scan is one of the most reliable methods for body composition analysis [67].

## 5. Conclusions

Mothers with a history of GDM had less favorable eating practices than mothers without prior GDM. More specifically, GDM+ mothers were more likely to prepare dinners using pre-prepared or processed foods, an eating practice associated with less favorable components of diet quality and altered anthropometric and cardiometabolic profiles. This study highlighted the importance of analyzing diet in a more comprehensive way among this high-risk population, focusing not only on the “what” but also on the “how” of eating. Future studies in this population should explore a broader range of eating practices and how they evolve from the complicated pregnancy through the years following delivery [68,69]. Given the higher risk of chronic diseases among GDM+ mothers and their offspring, the factors influencing cooking from scratch within this population should be further investigated.

## Figures and Tables

**Table 1:** Mothers’ characteristics according to history of gestational diabetes.

	GDM+ Mothers (*n* = 105)	GDM− Mothers (*n* = 38)	*p* *-* *Value*
Age (years)			0.034
20–29	1 (1.0)	4 (10.5)	
30–39	63 (61.2)	23 (60.5)	
40 or more	39 (37.9)	11 (29.0)	
Age of the youngest child			0.069
Preschool (0–5 years)	76 (73.8)	22 (57.9)	
School-age (6–12 years)	27 (26.2)	16 (42.1)	
Number of children			0.111
1 child	16 (15.4)	6 (15.8)	
2 children	64 (61.5)	18 (47.4)	
3 children	17 (16.4)	13 (34.2)	
4 children or more	7 (6.7)	1 (2.6)	
Household annual income (CAD/year)			0.433
0–39,999	12 (13.5)	7 (22.6)	
40,000–79,999	26 (29.2)	9 (29.0)	
80,000–99,999	22 (24.7)	4 (12.9)	
≥100,000	29 (32.6)	11 (35.5)	
Highest maternal level of education			0.652
High school or less	18 (18.4)	4 (12.5)	
CEGEP ^a^	21 (21.4)	6 (18.8)	
University	59 (60.2)	22 (68.8)	

Results are expressed as *n* (%). GDM+: mothers with a history of gestational diabetes; GDM−: mothers without a history of gestational diabetes. ^a^ In the Quebec education system, CEGEP refers to “Collège d’enseignement général et professionnel” and includes preuniversity programs and technical programs.

**Table 2:** Eating practices of mothers according to history of gestational diabetes.

	GDM+ Mothers (*n* = 105)	GDM− Mothers (*n* = 38)	*p-Value* ^1^	*p-Value* ^2^
Be mindful of your eating habits				
Number of meals in front of a screen, /day			0.436	0.353
<1	71 (72.5)	29 (76.3)		
≥1	27 (27.6)	9 (23.7)		
Eat meals with others				
Number of family meals, /day			0.435	0.328
≤2	71 (69.6)	29 (76.3)		
3	31 (30.4)	9 (23.7)		
Cook more often				
Dinners cooked from scratch, /week			0.621	0.480
<5	27 (26.5)	9 (23.7)		
≥5	75 (73.5)	29 (76.3)		
Dinners cooked with pre-prepared or processed foods, /week			0.016	0.006
None	52 (51.0)	25 (65.8)		
≥1	50 (49.0)	13 (34.2)		
Ready-to-eat dinners, /week			0.775	0.581
None	86 (84.3)	33 (86.8)		
≥1	16 (15.7)	5 (13.2)		
Dinners purchased at a take-out restaurant, /week			0.562	0.633
None	44 (43.1)	15 (39.5)		
≥1	58 (56.9)	23 (60.5)		

Results are expressed as *n* (%). GDM+: mothers with a history of gestational diabetes; GDM−: mothers without a history of gestational diabetes. ^1^ Adjustment for age, household annual income, highest maternal level of education, age of the youngest child, and number of children. ^2^ Further adjustment for energy intake and reporting status.

**Table 3:** Anthropometric and cardiometabolic profile of GDM+ mothers according to the number of dinners using pre-prepared or processed foods per week.

	No Dinner (*n* = 52)	≥1 Dinner (*n* = 50)	*p-Value* ^1^	*p-Value* ^2^
**Anthropometric profile**				
Total body fat (%)	34.0 ± 7.7	37.5 ± 7.6	0.041	0.090
Total android fat (%)	34.9 ± 12.7	40.2 ± 11.2	0.055	0.108
Waist circumference (cm)	87.1 ± 16.3	91.6 ± 13.9	0.030	0.088
Body mass index (kg/m^2^)	26.0 ± 7.1	27.6 ± 6.2	0.056	0.147
**Cardiometabolic profile**				
HOMA-IR ^a^	2.8 ± 1.8	2.8 ± 1.4	0.391	0.566
2 h plasma glucose ^b^ (mmol/L)	7.3 ± 2.6	7.3 ± 3.2	0.548	0.495
HbA1C ^c^ (%)	5.5 ± 0.3	5.6 ± 0.5	0.025	0.044
Triglycerides (mmol/L)	1.2 ± 0.6	1.1 ± 0.6	0.835	0.835
LDL cholesterol (mmol/L)	2.7 ± 0.7	2.5 ± 0.9	0.942	0.469
Cholesterol ratio ^d^	3.3 ± 1.0	3.1 ± 1.0	0.684	0.798

Results are expressed as mean ± SD. GDM+: mothers with a history of gestational diabetes. ^a^ HOMA-IR: homeostasis model assessment of insulin resistance. ^b^ Plasma glucose level after a 75 g, 2 h oral glucose tolerance test (OGTT). ^c^ HbA1C: hemoglobin A1c (glycated hemoglobin). ^d^ Cholesterol ratio: total cholesterol/HDL cholesterol. ^1^ Adjustment for age, household annual income, highest maternal level of education, moderate-to-vigorous physical activity, smoking and alcohol consumption. ^2^ Further adjustment for energy intake and reporting status.

**Table 4:** Anthropometric and cardiometabolic profile of GDM− mothers according to the number of dinners using pre-prepared or processed foods per week.

	No Dinner (*n* = 25)	≥1 Dinner (*n* = 13)	*p-Value* ^1^	*p-Value* ^2^
**Anthropometric profile**				
Total body fat (%)	32.1 ± 7.8	32.8 ± 7.4	0.089	0.313
Total android fat (%)	30.7 ± 13.0	32.2 ± 12.7	0.096	0.365
Waist circumference (cm)	80.4 ± 8.2	82.2 ± 9.8	0.162	0.617
Body mass index (kg/m^2^)	23.7 ± 4.0	24.4 ± 4.5	0.270	0.737
**Cardiometabolic profile**				
HOMA-IR ^a^	1.8 ± 0.7	2.1 ± 1.4	0.259	0.216
2 h plasma glucose ^b^ (mmol/L)	5.0 ± 1.4	5.2 ± 1.3	0.827	0.720
HbA1C ^c^ (%)	5.3 ± 0.3	5.4 ± 0.3	0.784	0.947
Triglycerides (mmol/L)	0.8 ± 0.3	0.9 ± 0.6	0.323	0.466
LDL cholesterol (mmol/L)	2.3 ± 0.7	2.6 ± 0.7	0.800	0.711
Cholesterol ratio ^d^	2.9 ± 0.7	3.0 ± 1.2	0.166	0.592

Results are expressed as mean ± SD. GDM−: mothers without a history of gestational diabetes. ^a^ HOMA-IR: homeostasis model assessment of insulin resistance. ^b^ Plasma glucose level after a 75 g, 2 h oral glucose tolerance test (OGTT). ^c^ HbA1C: hemoglobin A1c (glycated hemoglobin). ^d^ Cholesterol ratio: total cholesterol/HDL cholesterol. ^1^ Adjustment for age, household annual income, highest maternal level of education, moderate-to-vigorous physical activity, smoking and alcohol consumption. ^2^ Further adjustment for energy intake and reporting status.

## Data Availability

The data presented in this study are available upon request from the corresponding author, as they are not publicly available due to ethical restrictions.

## Supplementary Materials

The following supporting information can be downloaded at https://www.mdpi.com/article/10.3390/healthcare13212792/s1, Table S1: Energy intake and reporting status of GDM+ mothers according to the number of dinners using pre-prepared or processed foods per week. Table S2: Energy intake and reporting status of GDM− mothers according to the number of dinners using pre-prepared or processed foods per week. Table S3: Healthy Eating Food Index—2019 of GDM+ mothers according to the number of dinners using pre-prepared or processed foods per week. Table S4: Healthy Eating Food Index—2019 of GDM− mothers according to the number of dinners using pre-prepared or processed foods per week.

## Abbreviations

The following abbreviations are used in this manuscript:

BMI	Body mass index
CFG	Canada’s Food Guide
CVD	Cardiovascular diseases
DXA	dual-energy X-ray absorptiometry
FFQ	Food frequency questionnaire
GDM	Gestational diabetes mellitus
GDM+	History of gestational diabetes mellitus
GDM−	No history of gestational diabetes mellitus
GLMs	Generalized linear models
HbA1C	Glycated hemoglobin
HEFI-2019	Healthy Eating Food Index—2019
HOMA-IR	Homeostasis model assessment of insulin resistance
INAF	Institute of Nutrition and Functional Foods
NUTRISS	Centre Nutrition, Santé et Société
OGTT	Oral glucose tolerance test
T2D	Type 2 diabetes
