# Supplementary material for: Healthy Eating Is More than the Foods You Eat: Eating Practices of Mothers with and Without a History of Gestational Diabetes Mellitus
Source: Healthcare (Basel). 2025 Nov 4;13(21):2792. doi: 10.3390/healthcare13212792 (PMC12610001; doi:10.3390/healthcare13212792)
Supplement: Supplementary file 1 [file healthcare-13-02792-s001.zip › healthcare-3937499-supplementary.pdf]

Table S1. Energy intake and reporting status of GDM+ mothers according to the number of dinners using pre-prepared or processed foods per week

	**No Dinner (*n* = 52)**	**≥1 Dinner (*n* = 50)**	***p-Value***
Energy intake (kcal)	2099 ± 460	2287 ± 641	0.681*
Reporting status			0.386**
Under-reporters	8 (16.0)	6 (12.0)	
Plausible reporters	29 (58.0)	25 (50.0)	
Over-reporters	13 (26.0)	19 (38.0)	

Results are expressed as mean ± SD or *n* (%). GDM+: history of gestational diabetes. *Adjustment for age, weight, height, household annual income, highest maternal level of education, age of the youngest child, and number of children. **Adjustment for age, household annual income, highest maternal level of education, age of the youngest child, and number of children.

Table S2. Energy intake and reporting status of GDM- mothers according to the number of dinners using pre-prepared or processed foods per week

	**No Dinner (*n* = 25)**	**≥1 Dinner (*n* = 13)**	***p-Value***
Energy intake (kcal)	2404 ± 688	2737 ± 919	0.371*
Reporting status			0.030**
Under-reporters	2 (8.0)	0 (0.0)	
Plausible reporters	12 (48.0)	6 (46.2)	
Over-reporters	11 (44.0)	7 (53.9)	

Results are expressed as mean ± SD or *n* (%). GDM-: no history of gestational diabetes. *Adjustment for age, weight, height, household annual income, highest maternal level of education, age of the youngest child, and number of children. **Adjustment for age, household annual income, highest maternal level of education, age of the youngest child, and number of children.

Table S3. Healthy Eating Food Index - 2019 of GDM+ mothers according to the number of dinners using pre-prepared or processed foods per week

	**No Dinner (n = 48)**	**≥1 Dinner (n = 43)**	***p-Value*[1]**	***p-Value*[2]**
Total score (/80)	51.7 ± 8.5	49.1 ± 8.5	0.218	0.355
Vegetables and fruits (/20)	13.1 ± 3.8	13.4 ± 4.2	0.744	0.515
Whole-grain foods (/5)	1.9 ± 1.2	1.1 ± 0.8	0.002	0.004
Grain foods ratio (/5)	2.5 ± 1.3	2.0 ± 1.3	0.169	0.262
Protein foods (/5)	4.8 ± 0.5	4.9 ± 0.3	0.310	0.270
Plant-based protein foods (/5)	1.6 ± 1.1	1.4 ± 1.0	0.993	0.962
Beverages (/10)	8.7 ± 1.0	8.3 ± 1.4	0.285	0.256
Fatty acid ratio (/5)	2.1 ± 1.3	2.0 ± 1.4	0.923	0.833
Saturated fats* (/5)	2.6 ± 1.7	2.5 ± 1.8	0.968	0.850
Free sugars* (/10)	8.6 ± 2.8	8.5 ± 2.9	0.855	0.992
Sodium* (/10)	5.8 ± 2.0	4.9 ± 2.0	0.013	0.008

Results are expressed as mean ± SD. GDM+: history of gestational diabetes. The number in parentheses refers to the maximum score. *A higher score corresponds to a lower intake (moderation components). [1]Adjustment for age, household annual income, highest maternal level of education, age of the youngest child, and number of children. [2]Further adjustment for energy intake and reporting status.

Table S4. Healthy Eating Food Index - 2019 of GDM- mothers according to the number of dinners using pre-prepared or processed foods per week

	**No Dinner (n = 21)**	**≥1 Dinner (n = 12)**	***p-Value*[1]**	***p-Value*[2]**
Total score (/80)	52.6 ± 8.7	52.6 ± 6.0	0.324	0.537
Vegetables and fruits (/20)	14.5 ± 4.0	15.0 ± 1.9	0.783	0.995
Whole-grain foods (/5)	1.3 ± 1.2	1.6 ± 0.9	0.034	0.035
Grain foods ratio (/5)	2.1 ± 1.2	2.3 ± 1.2	0.055	0.057
Protein foods (/5)	4.9 ± 0.2	4.7 ± 0.4	0.891	0.704
Plant-based protein foods (/5)	1.6 ± 1.3	1.4 ± 0.9	0.157	0.564
Beverages (/10)	8.4 ± 0.9	7.9 ± 1.9	0.990	0.834
Fatty acid ratio (/5)	2.3 ± 1.4	2.2 ± 1.0	0.903	0.825
Saturated fats* (/5)	2.8 ± 1.9	3.1 ± 2.2	0.591	0.605
Free sugars* (/10)	8.9 ± 1.9	7.8 ± 2.7	0.520	0.998
Sodium* (/10)	6.0 ± 1.6	6.5 ± 1.7	0.755	0.687

Results are expressed as mean ± SD. GDM-: no history of gestational diabetes. The number in parentheses refers to the maximum score. *A higher score corresponds to a lower intake (moderation components). [1]Adjustment for age, household annual income, highest maternal level of education, age of the youngest child, and number of children. [2]Further adjustment for energy intake and reporting status.
